# The Utility of Sirolimus Eluting Balloons in the Setting of Chronic Limb Threatening Ischaemia in Asian Patients from Singapore – 12 Months Results of the PRISTINE Registry

**DOI:** 10.1007/s00270-024-03756-3

**Published:** 2024-06-19

**Authors:** T. Y. Tang, C. Yap, S. L. Chan, S. X. Y. Soon, C. Sivanathan, A. Gogna, A. K. Patel, T. T. Chong

**Affiliations:** 1 The Vascular & Endovascular Clinic, Gleneagles Medical Centre, Singapore, 258499 Singapore; 2 Department of Vascular Surgery, Singapore General Hospital, Singapore, Singapore; 3 Health Services Research Center, SingHealth, Singapore, Singapore; 4 Department of Vascular Interventional Radiology, Singapore General Hospital, Singapore, Singapore

**Keywords:** Chronic limb threatening ischaemia, Drug coated balloon, Limb salvage, Outcome, Percutaneous angioplasty, Selution SLR™, Sirolimus, Wound healing

## Abstract

**Purpose:**

The aim of *PRISTINE* was to evaluate the 6 and 12 months safety and efficacy of the *Selution Sustained Limus Release (SLR)*™ sirolimus-coated balloon for treatment of complex lower limb occlusive lesions (TASC II C & D) in patients with chronic limb threatening ischemia (CLTI) from Singapore.

**Methods:**

*PRISTINE* was a prospective, non-randomized, single arm, observational, multi-investigator, single-center clinical study. Complication-free survival at 30 days was the safety clinical endpoint. Immediate technical success (ability to cross and dilate the lesion and achieve residual angiographic stenosis < 30%), 6-month primary vessel patency, limb salvage, clinically driven target lesion revascularization (TLR) and amputation free survival (AFS) were the efficacy endpoints of interest.

**Results:**

Seventy five patients were included. There were 50 (68.0%) males; mean age, 69.0 ± 10.7 years. CLTI severity was based on the Rutherford Scale (R5 = 51; R6 = 17). Significant co-morbidities included diabetes mellitus (*n* = 68; 91.0%) and end-stage renal failure (*n* = 28; 37.0%). 112 atherosclerotic lesions were treated (TASC II D = 58 (52%); 76 (67%) de novo). There was 100% technical success. Mean lesion length treated was 22.4 ± 13.9 cm. Primary vessel patencies at 6 and 12 months were 64/86 (74%) and 43/74 (58%) and freedom from clinically driven TLR were 72/86 (84%) and 55/74 (74%) respectively. AFS was 61/73 (84.0%; five deaths and seven major lower extremity amputation) at 6-months. Mean Rutherford score improved from 5.1 ± 0.55 at baseline to 1.1 ± 2.05 (*p* < 0.05) at one year and there was a wound healing rate of 38/48 (79%) at the same timepoint.

**Conclusions:**

The *Selution SLR*™ drug eluting balloon is safe and efficacious in treating highly complex infra-inguinal atherosclerotic lesions in an otherwise challenging frail population of CLTI patients with a high incidence of diabetes and end-stage renal failure. It is associated with highly satisfactory acute technical and clinical success, 12-month target lesion patency and AFS.

**Level of Evidence:**

Level 2b, Individual Cohort Study.

**Graphical Abstract:**

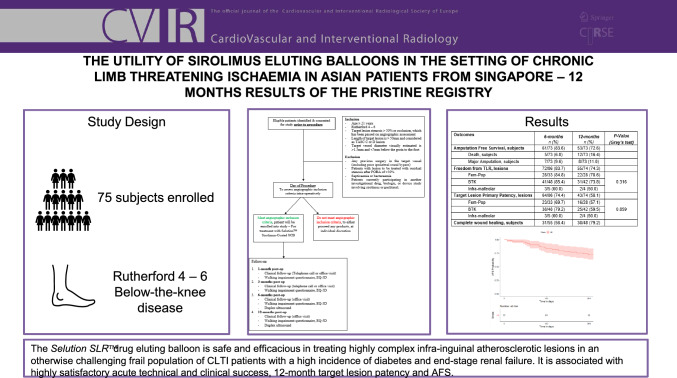

## Introduction

Chronic limb threatening ischemia (CLTI) represents the most advanced stage of peripheral of artery disease (PAD), associated with high major amputation rates and cardiovascular mortality.[1] In recent years, technical options for endovascular revascularization have advanced considerably, leading some to advocate for an “endovascular-first” approach for all patients with CLTI.[2] However, existing evidence supports a selective revascularization algorithm, dependent on specific clinical and anatomic scenarios.[2] Patients with CLTI usually present with multi-level infra-inguinal PAD and tibial arterial occlusions.[3] Despite initial high rates of technical success (> 90%), plain balloon angioplasty is mired by high rates of restenosis because of elastic recoil[4] and barotrauma caused by the intra-arterial ballooning process, which leads to neointimal hyperplasia (NIH).[5] NIH increases reinterventions and delays wound healing in CLTI patients, increasing morbidity and mortality.[6]

Devices coated with rapamycin analogues such as sirolimus have been used successfully to limit restenosis by inhibiting the biologic pathway that leads to NIH.[7] The effects of rapamycin analogues have been well-studied in the coronary circulation, where sirolimus-eluting stents have been shown to be safe and more effective than paclitaxel devices.[8] In the peripheral arterial arena, the use of sirolimus in the form of sirolimus eluting balloons (SEBs), has only begun to gain traction in the past few years. The published data for the use of SEBs in the peripheral vasculature remains limited to a handful of small pilot studies up to 12 months.[9–12] Our group has previously reported promising outcomes at 6 and 12 months from the PRESTIGE study using the *Selution Sustained Limus Release (SLR)*™ (M. A. MedAlliance SA, Nyon, Switzerland) SEB, for the treatment of complex tibial occlusive lesions (TASC II C & D) in patients with CLTI from Singapore.[9, 11] We found a sustained low target lesion revascularization rate (TLR) of 7.4%, high amputation-free survival (AFS) (84%) and complete wound healing rates (82%) through to one year. However, the use of SEBs were limited to a relatively small CLTI cohort (*n* = 25), elution only to the below knee tibial arteries and contribution of patients were from mainly a single operator.

The aim of the PRISTINE registry was to extend the use of this SEB to both the femoro-popliteal segments and the below knee arteries in the CLTI setting, to include a larger number of patients and whether it could replicate the PRESTIGE results using multi-investigators from the same institution with varying peripheral vascular interventional experience.

## Methods

### PRISTINE Study Design

In the **P**rospective **R**egistry to **I**nvestigate the **S**afety and efficacy of the **T**reatment with the SELUTION Sirolimus Drug Coated Balloon in TASC II C and D athero-occlusive **I**nfra-i**N**guinal disease in patients with chronic limb threatening ischemia from Singapor**E (PRISTINE)** (NCT04534257)*, Selution SLR™* was studied for the treatment of complex femoro-popliteal and tibial artery occlusive disease with TASC II C and D lesions in CLTI patients from a multi-ethnic Asian cohort from Singapore.

The study was conducted in accordance with good clinical practice standards and the ethical principles of the *Declaration of Helsinki* and its amendments. The local Institutional Review Board approved this study. Pre-morbid variables collected prospectively included patient demographics, co-morbidities, tissue loss severity based on the Rutherford Scale[13] and pre-operative toe pressure. Procedural data included TASC II lesion severity,[14] number of superficial femoral, popliteal and tibial arteries treated. Lesion calcification was graded according to the presence of radio-opacities within the arterial wall at the site of occlusion or stenosis as per Dattilo.[15]

The study design, with inclusion and exclusion criteria are similar to the PRESTIGE trial, which has previously been described.[11] The target was to recruit a total of 75 patients with CLTI. All patients received the SEB according to the following protocol. The target lesion was mandatorily predilated with conventional balloon and angiography performed to confirm that less than 30% residual stenosis was successfully achieved in the target lesion. SEB was then deployed by balloon inflation to rated burst pressure of 10 atmospheres for 2 min to maximise drug transfer to the arterial wall, in a 1:1 sizing to the conventional balloon. The number of SEBs used to treat the target lesions were not restricted. If two devices were required to cover the lesion, the specified balloon overlap was at least 10 mm. For patients with multilevel disease, treatment with SEB included both the femoropopliteal and tibial lesions. Angioplasty balloon diameters of 2–7 mm and 150 mm lengths were available for *PRISTINE*.

### Inclusion/Exclusion Criteria

Patients eligible for this study were ≥ 21 years of age with had a documented diagnosis of CLTI, presenting a score from 4 to 6 by Rutherford classification.[13] Angiographic inclusion criteria included de novo and post-PTA restenotic lesions located in the SFA/pop/tibial arteries suitable for endovascular therapy, target lesion length was > 50 mm and/or considered as TASC II C or D lesion according to the TASC II classification and the target lesion had angiographic evidence of stenosis > 50% or occlusion. Any patient with previous open vascular surgery of the index limb, undrained pus or spreading wet gangrene were excluded. The full eligibility criteria are as outlined in Table 1B.

**Table 1:** (A) Secondary outcome measures. (B)Inclusion and exclusion criteria

A. *Secondary Outcome Measures*
1. Primary Patency at 6- and 12-month follow-up; defined as absence of a hemodynamically significant stenosis on Duplex ultrasound at the target lesion and without Target Lesion Revascularization (TLR) within the time of procedure and the given follow-up
2. Technical success; defined as the ability to cross and dilate the lesion and achieve residual angiographic stenosis no greater than 30%
3. Freedom from clinically-driven TLR at 12-month follow-up; defined as a repeat intervention to maintain or re-establish patency within the region of the treated arterial vessel plus 5 mm proximal and distal to the treated lesion edge at the respective time points
4. Clinical success at follow-up; defined as improvement of Rutherford Classification at all follow-up time points of one class or more as compared to pre-procedure score
5. Wound Healing at 6 months defined as closure of the primary wound by more than 75%
6. Freedom from major target limb amputation within 6 and 12-months post-index procedure
B. *Inclusion Criteria*
1. Age of subject ≥ 21 years
2. Patient has CLTI with Rutherford 4 – 6
3. Patient understand nature of procedure and provides written informed consent prior to enrolment
4. Willing to comply with specified follow-up evaluation
5. Projected life expectancy of at least 12 months and has not suffered an Myocardial Infarction within past 30 days
*Angiographic Inclusion Criteria*
6. De novo and post-PTA restenotic lesions located in the SFA/pop/tibial arteries suitable for endovascular therapy
7. Target lesions length > 50 mm and considered TASC C or D lesions according to TASC II classification
8. Target lesions has angiographic evidence of > 50% stenosis or occlusion, which had been passed with standard guidewire manipulation and predilated to < 30% residual stenosis using prolonged CBA (3 min) prior to enrolment
9. Target vessel diameter visually estimated to be > 1.5 mm and < 7 mm below to the groin to foot
10. Lesions in treated segment may be continuous or may have gaps present between stenosis and occlusions
11. Tibial vessels intervened on must have distal reconstitution above ankle (both patent inflow and ≥ 1 outflow extending to below ankle)
12. Inflow iliac lesions can be treated during the same procedure (must have < 30% residual stenosis and no evidence of embolization prior to treatment below groin)
*Exclusion Criteria*
1. Previous open vascular surgery
2. Permanently wheelchair bound or bedridden
3. Presence of a stent that was placed during a previous procedure
4. Intervention performed in preparation for a planned major amputation
5. Untreated flow-limiting inflow lesions
6. Patients for whom antiplatelet therapy, anticoagulants or thrombolytic drugs are contraindicated
7. Patients with uncorrected bleeding disorder
8. Untreatable lesion at distal outflow arteries
9. Aneurysm at SFA/Popliteal artery
10. Non-atherosclerotic disease resulting in occlusion (e.g. embolism, Buerger’s Disease, Vasculitis)
11. Any condition preventing patient from complying with the study protocol if patient has a life expectancy of < 1 year
12. Major distal amputation (above the transmetatarsal) in study limb or non-study limb
13. Septicaemia or Bacteraemia
14. Undrained pus or spreading wet gangrene in foot that is not controlled at the time of revascularization procedure
15. Neurotropic ulcer or heel pressure ulcer or ulcer potentially involving calcaneus
16. Episode of acute limb ischaemia within the previous 1 month
17. Use of atherectomy or laser devices during procedure
18. Known hypersensitivity or contraindication to contrast media, heparin and sirolimus
19. Immunosuppressed patients

### Outcome Measures

Clinical primary safety outcome measure was freedom from major adverse clinical events (MACE), a composite of freedom from device- and procedure-related mortality through 30 days. Performance primary outcome measure was freedom from clinically driven TLR within 6 and 12 months post-index procedure. Clinically driven TLR was defined as any re-intervention performed for ≥ 50% diameter stenosis (on Duplex ultrasound) at the target lesion after documentation of recurrent or continuing clinical symptoms of the patient. Secondary outcome measures are as outlined in Table 1A.

### Follow-up

**Fig. 1:**
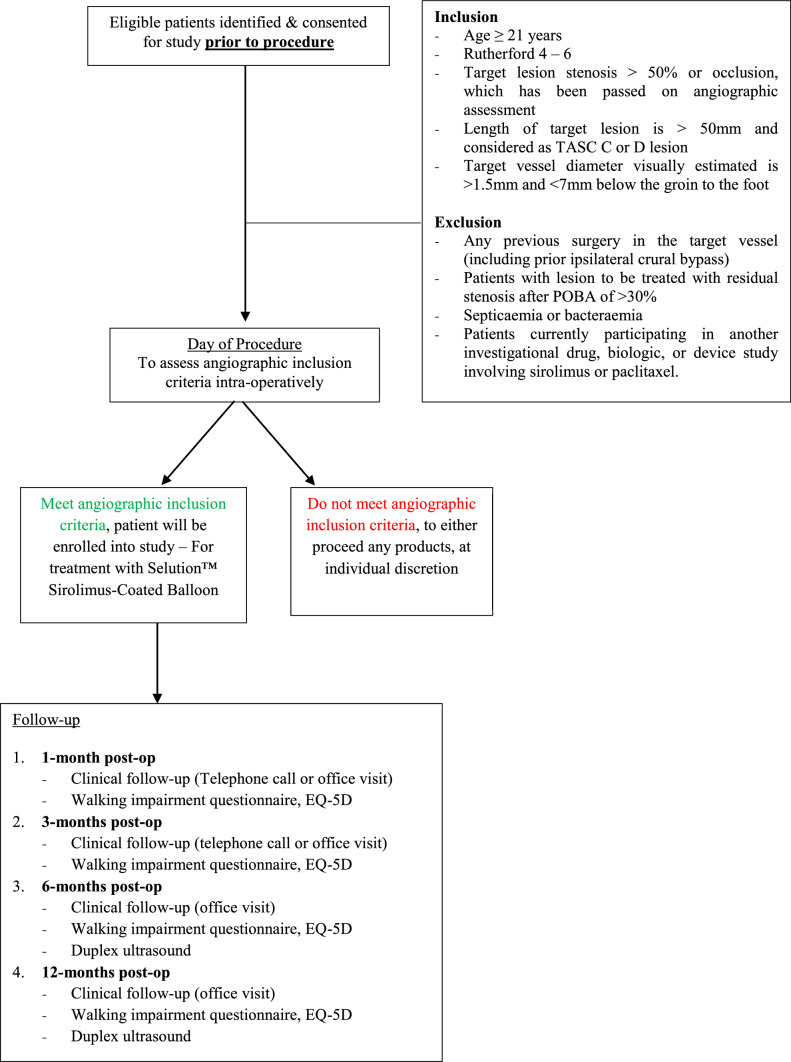
PRISTINE Trial flow diagram

Clinical assessments were performed at 1, 3 and 6 months to assess clinical progress and wound healing. At each follow-up, Walking Impairment Questionnaire (WIQ) and EuroQol-5 Dimension (EQ5D) was done to measure improvement in quality of life. There was a mandatory 6-month and 12-month Duplex ultrasound to check artery patency (Fig. 1).

### Statistical Analysis

Baseline variables were summarised with the use of descriptive statistics. Continuous variables were reported as the mean and standard deviation, or median and range, as appropriate, and categorical variables as absolute number and percent. For TLR, amputation of the same limb or death were competing events that would preclude TLR, therefore the cumulative incidence of TLR was estimated from competing risks data using the *cmprsk* package in R. For AFS and death, the survival probability was estimated using Kaplan Meier analysis. All analyses were performed in R version 3.5.1.[16]

## Results

### Patient Demographics

Seventy five patients (54(72%) Chinese in origin) were enrolled over an 8-month period. 50/75 (66.7%) were male and mean age was 69.0 ± 10.7 years. 68/75(90.7%) had diabetes mellitus and 28/75 (37.3%) had end-stage renal failure (ESRF). 68/75 (90.7%) patients had some degree of tissue loss (Rutherford category 5 = 51/75(68.0%), Rutherford category 6 = 17/75(22.7%)) and mean presenting WIFI[17] score was 4.07 ± 1.43. Using the *Society for Vascular Surgery* clinical stages by expert consensus,[17] 51/75(68.0%) limbs were at high or moderate risk of major amputation at 1-year. Table 2 shows the subject demographics.

**Table 2:** Patient Demographics

	PRISTINE (n = 75)
Mean Age, years (SD)	69.0 ± 10.7
Mean BMI (SD)	24.3 ± 3.97
**Gender**	
Male	50 (66.7)
Female	25 (33.3)
**Ethnic Group**	
Chinese	54 (72.0)
Malay	10 (13.3)
Indian	8 (10.7)
Others	3 (4.0)
**Smoking status**	
Non-smoker	48 (64.0)
Smoker	15 (20.0)
Ex-smoker	12 (16.0)
**Co-Morbidities (%)**	
Diabetes	68 (90.7)
Hypercholesterolemia	65 (86.7)
Hypertension	67 (89.3)
Cerebrovascular accident	2 (2.7)
MI	24 (32.0)
End Stage Renal Failure (ESRF)	28 (37.3)
**Medication History**	
Aspirin	58 (77.3)
Clopidogrel	16 (21.3)
Anti-coagulants	9 (12.0)
Statin	72 (96.0)
ACE Inhibitor	16 (21.3)
Angiotensin Receptor Antagonist	28 (37.3)
Beta-blocker	45 (60.0)
Insulin	39 (52.0)
Diabetic Medication	47 (62.7)
**Location of index wound**	
Digit	44 (64.7)
Forefoot	6 (8.8)
Heel	6 (8.8)
Shin	3 (4.4)
Dorsum	5 (7.4)
Plantar	4 (5.9)
Average Toe Pressure (mmHg), SD	43.4 ± 23.8
**Rutherford Classification**	
**4** *(Rest pain)*	7 (9.3)
**5** (*Minor tissue loss – nonhealing ulcer, focal gangrene with diffuse pedal ischema*)	51 (68.0)
**6** *(Major tissue loss – Severe ischemic ulcers or gangrene)*	17 (22.7)
**Average WIfI Score**	4.07 ± 1.43

### Lesion Characteristics

Seventy five legs (112 infrainguinal lesions; 76 de novo and 36 restenosis) were treated. 58/112(51.8%) were TASC D lesions. Mean lesion length treated was 22.4 ± 13.9 cm. The anterior tibial artery (ATA) was the most treated tibial vessel (28/112(25.0%)). There was a high incidence of moderate to severe vessel wall calcification (99/112(88.4%)). Majority of patients (48/75;64.0%) had multi-level atherosclerotic disease below the groin. Infra-malleolar disease was treated in (12/75,16.0%) limbs. Table 3 shows target lesion characteristics and procedural data.

**Table 3:** Lesion Details

Characteristics	n (%)
**Operative Details**	
**Leg treated**	
Left	39 (52.0)
Right	36 (48.0)
Number of vessel runoff post-angioplasty	
1	29 (38.7)
2	31 (41.3)
3	15 (20.0)
Concomitant treatment of infra-malleolar disease	12 (16.0)
**Lesion-specific details (n = 112)**	
Location of Treated Vessel (n = 112)	
Superficial Femoral Artery (SFA)	17 (15.2)
Popliteal	6 (5.4)
Femoro-popliteal	20 (17.9)
Anterior Tibial Artery (ATA)	28 (25.0)
Posterior Tibial Artery (PTA)	21 (18.8)
Peroneal	7 (6.3)
TP Trunk	4 (3.6)
Common Plantar Artery	4 (3.6)
Dorsalis Pedis Artery (DPA)	5 (4.5)
De novo lesions	76 (67.9)
Re-stenotic lesions	36 (32.1)
TASC Classification	
C	54 (48.2)
D	58 (51.8)
Lesion Length (mm), mean ± sd	224.3 ± 139.1
Diameter Stenosis (%), mean ± sd	84.1 ± 15.0
Bailout Stenting	8 (7.1)
Calcification Classification	
1 (None)	2 (1.8)
2 (focal)	4 (3.6)
3 (mild)	7 (6.3)
4 (moderate)	54 (48.2)
5 (severe)	45 (40.2)

### Outcomes

There was 100% technical success. There were no retained device components events or device-related deaths. There was no balloon slippage during inflation. No device-related serious adverse events were reported during the first 30 days.

**Fig. 2:**
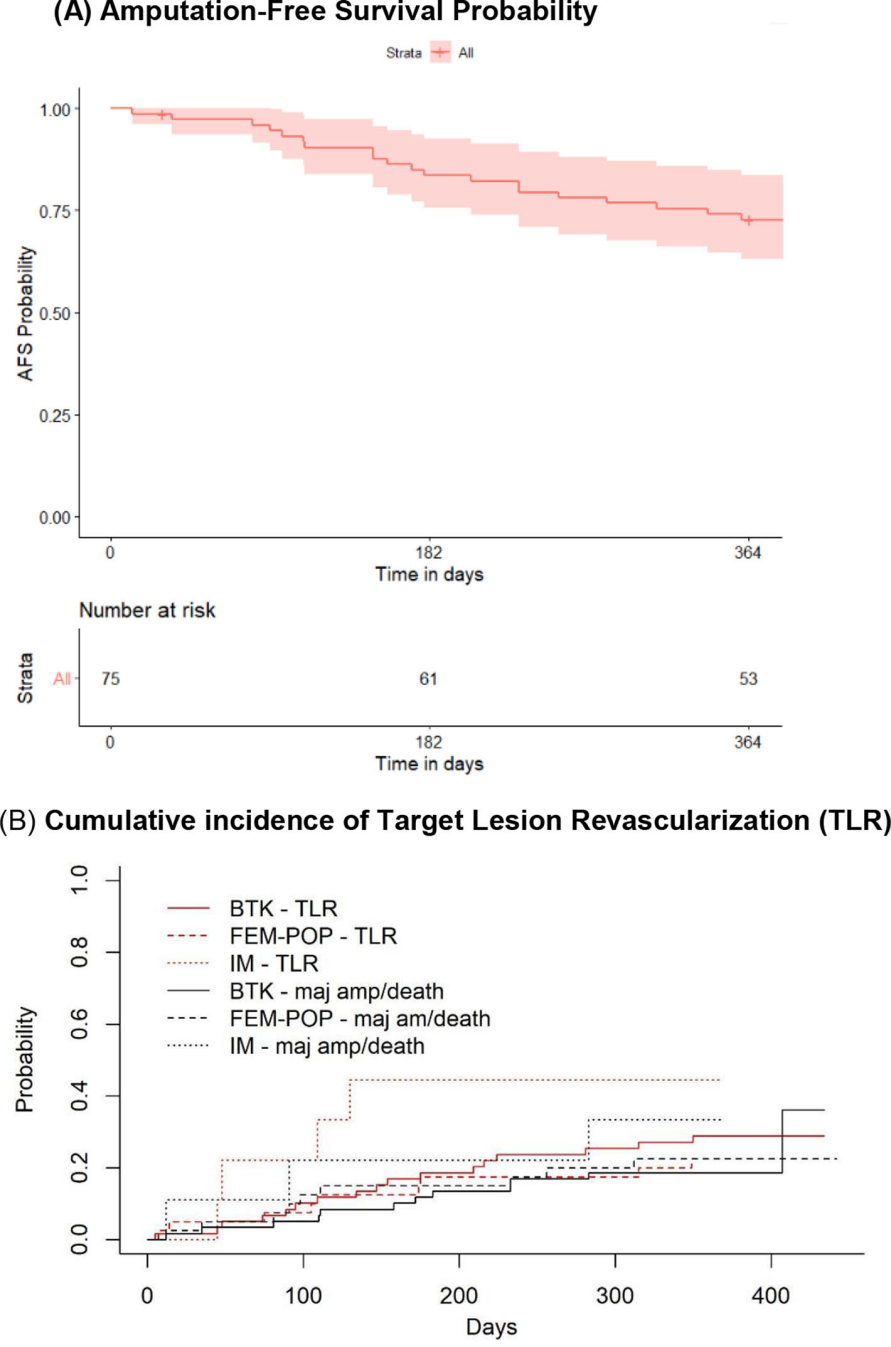
(A) AFS probability estimated using Kaplan Meier analysis. (B) Cumulative incidence of TLR events estimated from competing events that preclude TLR I.e. amputation and death

**Fig. 3:**
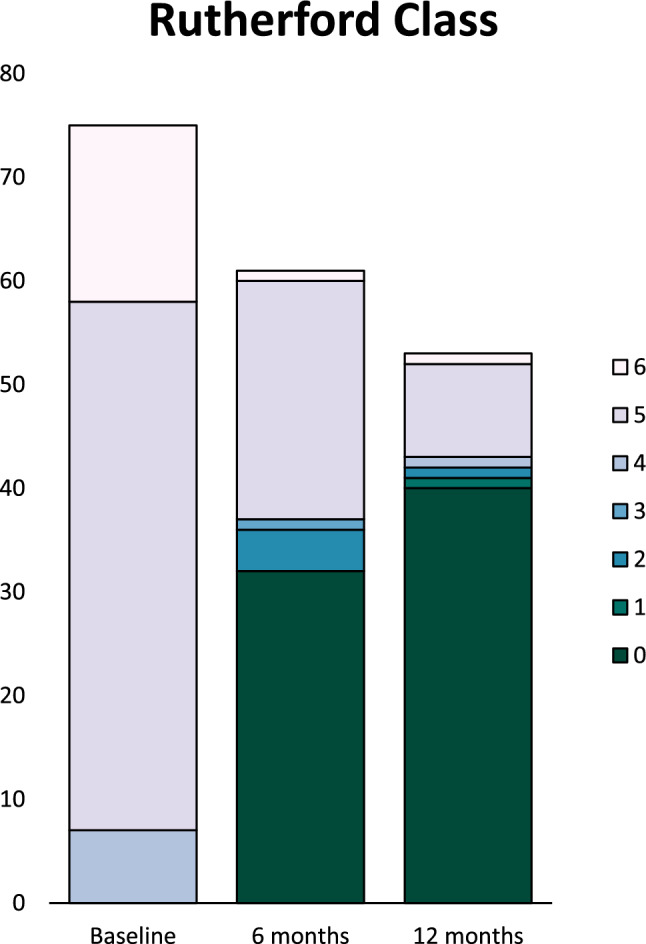
Rutherford classification at baseline, 6 months and 12 months. [From 51/75 (68.0%) subjects assessed as Rutherford 5 at baseline, the percentage decreases to 37.7% at 6 months and finally 17.0% at 12 months. 44/53 (83.0%) of subjects have shown improvement by at least 1 category by 12 months]

**Fig. 4:**
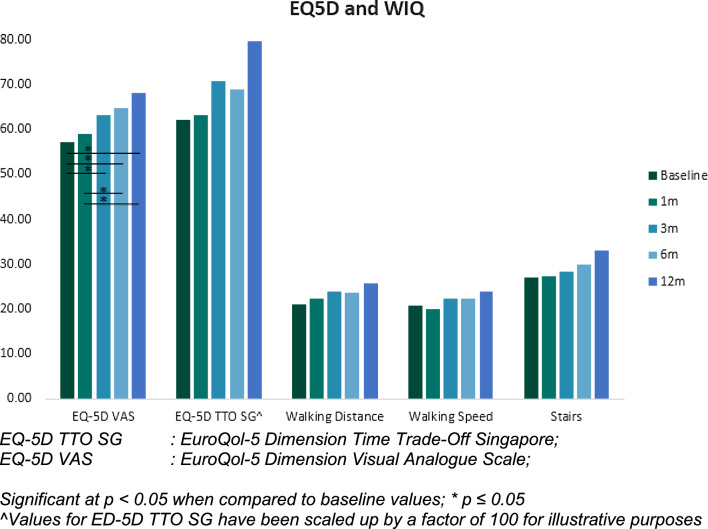
EQ5D and walking impairment questionnaire

Target Lesion Primary Patency (TLPP) at 6 months was 64/86(74.4%) and freedom from clinically driven TLR was 72/86 (83.7%). AFS at 6 months was 61/73(83.6%) (five deaths and seven major lower extremity amputation). Two subjects were lost to follow-up. TLPP at 12 months was 43/74(58.1%) and the freedom from clinically driven TLR was 55/74(74.3%). AFS was 53/73(72.6%) (Table 4). Amputation-free survival probability and cumulative incidence of TLR and are presented in Figs. 2A, B respectively. The mean Rutherford score at baseline was 5.13 ± 0.55 and improved to 2.16 ± 2.42 and 1.09 ± 2.05 at 6 and 12 months respectively. There was a wound healing rate of 31/55(56.4%) and 38/48(79.2%) at 6 and 12 months respectively. 44/53(83.0%) patients showed improvement by at least 1 Rutherford category by 12 months (Fig. 3). There was a significant improvement in the EQ5D VAS Scores at 3, 6 and 12 months compared to baseline (Fig. 4) which may be related to wound healing and regaining independence to ambulate again.

**Table 4:** Summary of Outcomes

**Outcomes**	***6-months*** *n (%)*	***12-months*** *n (%)*	***P-Value*** ***(Gray’s test)***
**Amputation Free Survival, subjects**	61/73 (83.6)	53/73 (72.6)	
Death, subjects	5/73 (6.8)	12/73 (16.4)	
Major Amputation, subjects	7/73 (9.6)	8/73 (11.0)	
**Freedom from TLR, lesions**	72/86 (83.7)	55/74 (74.3)	
Fem-Pop	28/33 (84.8)	22/28 (78.6)	0.316
BTK	41/48 (85.4)	31/42 (73.8)	
Infra-malleolar	3/5 (60.0)	2/4 (50.0)	
**Target Lesion Primary Patency, lesions**	64/86 (74.4)	43/74 (58.1)	
Fem-Pop	23/33 (69.7)	16/28 (57.1)	0.859
BTK	38/48 (79.2)	25/42 (59.5)	
Infra-malleolar	3/5 (60.0)	2/4 (50.0)	
**Complete wound healing, subjects**	31/55 (56.4)	38/48 (79.2)	

BTK: Below-the-knee, TLR: Target Lesion Revascularization

## Discussion

The PRISTINE registry is representative of everyday lower limb intervention practice for CLTI Asian patients in Singapore, who have long complex occlusive atherosclerotic lesions (mean of > 20 cm length) and heavy wall calcification. The data are relatively non-sanitized compared to RCTs, with liberal inclusion and few exclusion criteria. The strengths of the registry are that the follow-up of patients has been tight and prospective in nature with only few dropouts. The results demonstrated no peri-procedural early safety concerns, with no device complications or serious adverse events attributable to the device through to one year. It showed promising efficacy and clinical outcomes at 6 and 12 months, similar to other recent publications evaluating the performance of current SEBs on the market.[9, 11, 12] Despite high rates of diabetes mellitus, ESRF and moderate/severe vessel wall calcification, we have shown a 100% technical success rate (100%) and satisfactory 6- and 12- month primary vessel patencies of 74% and 58% respectively and freedom from clinically driven TLR of 84% (6 months) and 74% (1 year). An AFS of 84% and mortality rate of 7% at 6 months and 16% at 1 year are in keeping with what is expected from a challenging, frail population of patients with multiple co-morbidities.12-month primary patency rates for below-the-knee lesions and all-cause mortality rates were comparable to those reported in *Stefanos *et al.*’s* meta-analysis (59% vs 64% and 16% vs 9.0%, respectively). However, 12-month primary patency rates for above-the-knee lesions were lower in our cohort (57% vs 82.0%).[18] The SFA/pop subgroup is interesting in that the target lesion primary patency is lower than expected but fTLR is much higher. This may reflect SGH’s high threshold appetite for stenting an otherwise small Asian SFA/popliteal diseased artery because of concerns of stent complications.[19] The primary patency for these lesions were also lower compared to the XTOSI cohort of patients at both at 6 months (74% vs 88%) and 12 months (58% vs 79%).[12] XTOSI enrolled Asian CLTI patients with both fem-pop and BTK arterial lesions and used the MagicTouch PTA SEB (Concept Medical Inc., Florida, US), although their reporting definition was per patient rather than per lesion and the study included patients with intermittent claudication with less severe disease and co-morbidities on presentation. For BTK disease, the 6-month primary patency of 74% was comparable to MagicTouch.

A meta-analysis by *Katsanos *et al. reported significantly lower AFS in the paclitaxel-coated balloon arm as compared to plain balloon angioplasty, especially when high dose DCBs (3.0–3.5 μg/mm2) were used.[20] This was postulated to be due to distal non-target paclitaxel embolization resulting in micro-vessel occlusion, critical especially in the CLTI setting with minimal arterial reserve in the foot. One issue pertaining to the use of paclitaxel-based DCBs is the potential for the slow-flow phenomenon.[21] This was thought to be due to particulate embolization with the application of DCB with over 50% of drug lost down-stream reported.[22] This was thought to be a possible cause for the poorer outcomes with paclitaxel devices use reported in the meta-analyses by Katsanos *et al*.[23, 24] Early studies suggest no evidence of slow-flow phenomenon with the use of SEBs in the treatment of PAD, even for treatment of below ankle disease.[11] This was attributable to the micro-reservoirs of phospholipid polymer complex with the cell adherent technology of the Selution SLR™ SEB, which minimizes distal embolization.[21] Significant wound healing progress and improvement in ambulatory symptoms occurred with the balloon through 12 months. A complete wound healing rate of 79% is comparable, if not superior, with an open bypass salvage strategy [25] and previously published data on the use of paclitaxel coated balloons rates in lower limb endovascular revascularization for tissue loss.[26]

The results of PRISTINE may not be as impressive as those from PRESTIGE at the same time points, but this may be explained by the fact that PRESTIGE only included Rutherford 5 wounds whereas in PRISTINE, 22% had Rutherford 6 type advanced wounds and more than 50% of target lesions were TASC II D in nature. Furthermore, PRESTIGE only included tibial arterial lesions, whereas both femoro-popliteal and below the knee disease were allowed for PRISTINE, making the likelihood of mutli-level disease more likely to be treated with its intendent risks of restenosis and reintervention. While the initial results are encouraging and long-term follow-up is ongoing, the PRESTIGE Trial did not have a full range of SEB balloon sizes available to treat the entire vascular bed. PRISTINE included at least twice the number of patients to PRESTIGE, had the variable of multiple operators with differing peripheral vascular intervention experience using the technology and one of the aims was to determine whether complete revascularization of the affected vessel beds with SEBs will improve clinical outcomes as compared to treatment of only the tibial arteries. The results are encouraging in that they can reproduce similar results to PRESTIGE albeit with a bigger sample cohort with multiple proceduralists involved.

### Limitations

PRISTINE’s limitations were its single arm, single centre, non-randomized nature and relatively small sample size with a self-adjudicated design. There was no comparator arm to draw to. No quantitative angiographic analysis was performed to look at exact lesion lengths, reference vessel diameters and luminal gain post angioplasty for each target lesion. Furthermore, there was no core lab adjudication for artery patency at follow-up and Duplex ultrasound was the modality of choice to assess TLPP, with its inherent limitation of operator bias. Furthermore, Duplex follow up was not core lab adjudicated. Whilst convenient, there is also no consensus to accurately determine vessel patency using Duplex ultrasound for BTK lesions.

Although this registry did not raise any early safety concerns, the data presented is through to one year only. Together with the small number of patients included, no conclusions can yet be made regarding the long term safety of SEBs. Another limitation was the attrition rate for primary patency assessments at 6 and 12 months; although mortality and major lower limb amputations were the main reasons for the drop out, which are expected for this type of frail cohort with multiple co-morbidities.

Furthermore, the wound care practiced at SGH for CLTI diabetic foot is relatively unique whereby the wound debridement and post-operative care are performed by the vascular specialist themselves in a timely manner with all the latest wound products and technology available to promote tissue granulation, which may limit the generalizability of our results.

## Conclusions

Data from the PRISTINE registry show that the *Selution SLR*™ SEB is safe to use at least in the short term and efficacious in treating both above-the-knee and below-the-knee lesions, in a challenging cohort of Asian CLTI patients who have long complex occlusive atherosclerotic lesions and heavy wall calcification. It was associated with favorable 6 and 12-month primary patencies, freedom from clinical target lesion revascularization and foot wound-healing rates. Further studies are required to confirm these results in a larger cohort of patients in a RCT setting.
